# Stereotactic body radiotherapy versus conventional radiotherapy for painful bone metastases: a systematic review and meta-analysis of randomised controlled trials

**DOI:** 10.1186/s13014-022-02128-w

**Published:** 2022-09-13

**Authors:** Kei Ito, Tetsuo Saito, Naoki Nakamura, Nobuki Imano, Peter Hoskin

**Affiliations:** 1 Division of Radiation Oncology, Department of Radiology, Tokyo Metropolitan Cancer and Infectious Diseases Center Komagome Hospital, 3-18-22 Honkomagome, Bunkyo-ku, Tokyo, 113-8677 Japan; 2 Department of Radiation Oncology, Arao Municipal Hospital, 2600, Arao, Arao-shi, Kumamoto, 864-0041 Japan; 3 Department of Radiation Oncology, St. Marianna University School of Medicine, 2-16-1 Sugao, Miyamae Ward, Kawasaki, Kanagawa 216-8511 Japan; 4 Department of Radiation Oncology, Graduate School of Biomedical and Health Sciences, Hiroshima University, 1-2-3 Kasumi, Minami Ward, Hiroshima, 734-8551 Japan; 5 Mount Vernon Cancer Centre, Rickmansworth Rd, Northwood, HA6 2RN UK; 6 Division of Cancer Sciences, University of Manchester, 604 E College Ave, North Manchester, 46962 UK

**Keywords:** Metastasis, Stereotactic body radiotherapy, Randomised controlled trial, Systematic review, Meta-analysis, Quality of life

## Abstract

**Background:**

Stereotactic body radiotherapy (SBRT) is a promising approach in treating painful bone metastases. However, the superiority of SBRT over conventional external beam radiotherapy (cEBRT) remains controversial. Therefore, this systematic review and meta-analysis of randomised controlled trials was conducted to compare SBRT and cEBRT for the treatment of bone metastases.

**Methods:**

A search was conducted using PubMed on January 22, 2022, with the following inclusion criteria: (i) randomised controlled trials comparing SBRT with cEBRT for bone metastases and (ii) endpoint including pain response. Effect sizes across studies were pooled using random-effects models in a meta-analysis of risk ratios.

**Results:**

A total of 1246 articles were screened, with 7 articles comprising 964 patients (522 and 442 patients in the SBRT and cEBRT arms, respectively) meeting the inclusion criteria. The overall pain response (OR) rates of bone metastases at 3 months were 45% and 36% in the SBRT and cEBRT arms, respectively. The present analyses showed no significant difference between the two groups. In four studies included for the calculation of OR rates of spinal metastases at three months, the OR rates were 40% and 35% in the SBRT and cEBRT arms, respectively, with no significant difference between the two groups. The incidence of severe adverse effects and health-related quality of life outcomes were comparable between the two arms.

**Conclusions:**

The superiority of SBRT over cEBRT for pain palliation in bone metastases was not confirmed in this meta-analysis. Although SBRT is a standard of care for bone metastases, patients receiving SBRT should be selected appropriately.

**Supplementary Information:**

The online version contains supplementary material available at 10.1186/s13014-022-02128-w.

## Background

Conventional external beam radiotherapy (cEBRT) remains the standard of care for the palliative management of painful bone metastases [1]. Multiple phase III trials and meta-analyses have proven its palliative efficacy with few adverse events (AEs) regardless of the dose fraction schedules, including 8 Gy in a single fraction, 20 Gy in five fractions, and 30 Gy in ten fractions [2, 3]. However, cEBRT is not always effective, with published data showing overall pain response (OR) and complete pain response (CR) rates of approximately 60% and 25%, respectively [3, 4], and short net pain relief (i.e., patients whose pain improved after cEBRT experienced improvement for only 56.6% of their remaining lives) [5].

Stereotactic body radiotherapy (SBRT) is a high-precision radiotherapy technique that delivers an ablative biological dose in a few high-dose fractions while sparing adjacent risk organs [6]. SBRT may have an advantage over cEBRT in patients who can benefit from high-dose radiation. Many retrospective reports and single-arm phase II trials of SBRT for painful bone metastases have shown excellent outcomes [7–9]. However, two phase III trials comparing the pain relief effects of SBRT and cEBRT yielded conflicting results [10, 11]. Hence, the superiority of SBRT over cEBRT remains controversial. This systematic review and meta-analysis of randomised phase II and III trials was conducted to determine whether SBRT is superior to cEBRT for pain relief in bone metastases.

## Methods

### Data sources and study selection

The study protocol was approved by the institutional ethical review board of Tokyo Metropolitan Cancer and Infectious Diseases Center Komagome Hospital (approval number: 2844). This review was reported according to the Preferred Reporting Items for Systematic Reviews and Meta-analyses guidelines [12]. No formalised review protocol was created or registered to a database. The PubMed database was searched for relevant publications on January 22, 2022, irrespective of the publication date. The full search strategy is presented in Additional file 1. We contacted the study authors to collect missing data for the synthetic analysis. Studies that met the following inclusion criteria were included in the present meta-analysis: (i) randomised controlled trials (RCTs) comparing SBRT with cEBRT for bone metastases, (ii) endpoint including pain response, and (iii) a sample size of ≥ 10 patients in each arm. SBRT was defined based on the Canadian Association of Radiation Oncology task force as follows: the precise delivery of highly conformal and image-guided hypofractionated external beam radiotherapy to an extracranial body target in a single or few fractions of doses at least biologically equivalent to a radical course [6]. In contrast, cEBRT was defined as palliative radiotherapy using dose fraction schedules recommended by the American Society for Radiation Oncology (ASTRO) (a single 8 Gy fraction, 20 Gy in five fractions, 24 Gy in six fractions, and 30 Gy in ten fractions) [1] and conventional irradiation techniques. Two reviewers (K.I. and N.I.) independently performed the systematic review and a full-text review.

### Data extraction and risk of bias assessment

The primary outcome measure of interest was the OR rate for pain from bone metastases at 3 months in an intention-to-treat (ITT) analysis. For synthetic analysis, if the pain response could not be evaluated at 3 months, it was recorded at a time as close to 3 months as possible. The secondary outcomes in the ITT analysis included the OR rate at 3 months of only evaluable patients, the CR rate at 3 months, the OR rate at 6 months, AEs (such as pain flares, pathological fractures, and neurological injuries), and health-related quality of life (QOL). In addition, the above analyses on pain response were also performed in spinal metastases as a subgroup analysis. Data were extracted and independently reviewed by two authors (K.I. and N.I.). The risk of bias in the included studies was assessed by these two authors independently using the Cochrane risk of bias tool [13], and disagreements were addressed by discussion.

### Statistical analysis

Statistical analysis was performed using R Statistical Software (version 4.1.2; R Foundation for Statistical Computing, Vienna, Austria). Pooling of effect sizes across studies was conducted using random-effects models in a meta-analysis of risk ratios (RRs). The weights of the studies were calculated using the Mantel–Haenszel method without continuity correction. RRs and 95% confidence intervals (CIs) for each study were represented by forest plots. The heterogeneity of the included studies was assessed using the Cochran’s Q test and *I*^*2*^ index [14]. Random-effects logistic models were used to estimate the pooled response rates for the SBRT and cEBRT groups. Publication bias was assessed by inspecting a funnel plot of the ITT analysis of the overall response rates at 3 months. Statistical significance was set at p < 0.05.

## Results

### Characteristics of included studies

**Fig. 1:**
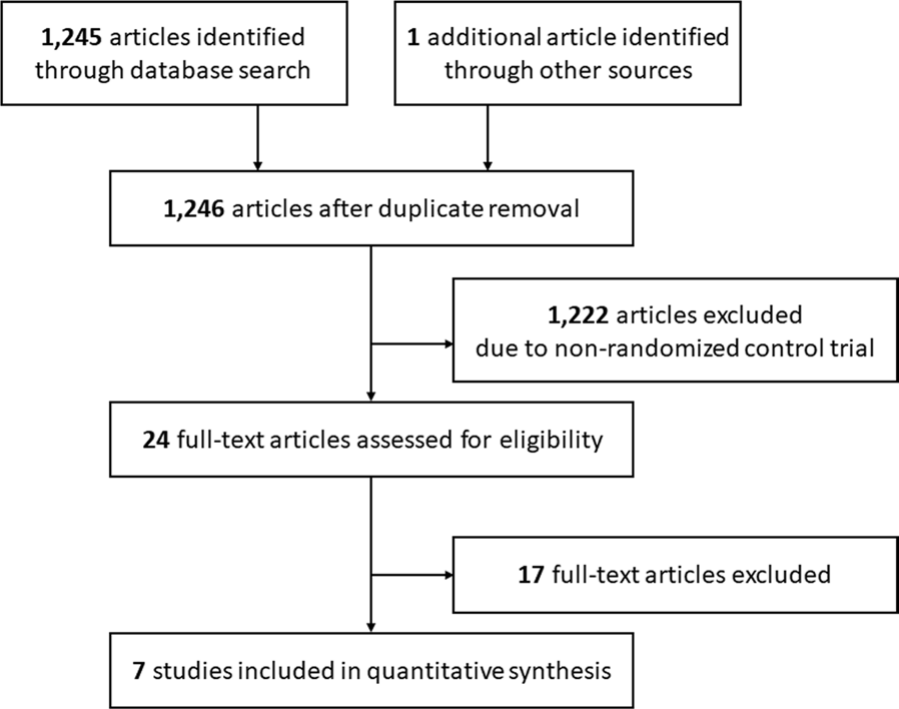
Flow diagram of search strategy

A total of 1245 articles were initially selected, and one abstract presented at the 2019 ASTRO annual meeting was added to the list. Among these, 1222 articles were excluded because they were non-RCTs. Thus, 24 publications were identified for full-text review, 17 of which were excluded based on the abovementioned criteria. Finally, seven studies comprising 964 patients that met all the inclusion criteria were included in this study (Fig. 1 and Additional file 1) [10, 11, 15–19]. For the RTOG0631 trial reported by Ryu et al. [10], only the abstract and presentation slides from the 2019 ASTRO annual meeting were available. Detailed information on AEs in the RTOG0631 trial was obtained from a clinical trial registry database [20].

The characteristics of the included studies are summarised in Table 1. The seven included studies comprised two phase III and five phase II trials. There were three trials involving spinal metastases, one involving non-spine bone metastases, and three involving both. One trial was a three-arm randomised study [16], but one arm (radiotherapy of a single 8 Gy fraction with an escalated dose to the central part of the target) was excluded because the treatment did not satisfy the cEBRT or SBRT definitions used in the present study. Although the prescribed SBRT dose varied greatly in each trial, 82.8% (432/522) of patients received SBRT at a biological equivalent dose (BED) of 40–60 Gy (α/β = 10). All but one trial evaluated pain response according to the criteria defined by the International Consensus Pain Response Endpoints [21] (one trial [10] defined partial response as an improvement of ≥ 3 points without increasing the analgesic dose). One trial evaluated pain response only after one month [16]. Hence, the 1-month data were used to analyse the 3-month OR rate in that trial. The number of patients with pain response at 6 months in the RTOG0631 trial were estimated based on the OR rate and the number of patients who answered the Brief Pain Inventory [10].

**Table 1:** Characteristics of included studies listed in the ascending order of the BED dose

Author	Year published	Study design	No. of patients	No. of patients with spinal metastases	Minimum pain score for entry	cEBRT dose (Gy/fx)	SBRT dose (Gy/fx)	SBRT dose (BED_10_, Gy)	Dose gradient inside the PTV in SBRT	Partial response definition	Complete response definition
Nguyen et al. [15]	2019	Phase 2	160	0	2	30/10	12/1, 16/1	26.4, 41.6	PTV Dmax < 115% PD	ICPRE criteria	ICPRE criteria
Berwouts et al. [16]	2015	Phase 2	30	12	2	8/1	16/1	41.6	PTV Dmax ≤ 112.5% PD	ICPRE criteria	ICPRE criteria
Ryu et al. [10]	2019	Phase 3	353	353	5	8/1	16/1, 18/1	41.6, 50.4	No rules	Improvement of ≥ 3 points without increasing the analgesic dose	ICPRE criteria
Sakr et al. [17]	2020	Phase 2	22	Not available	4	20/5	27/3	51.3	Not available	ICPRE criteria	ICPRE criteria
Sahgal et al. [11]	2021	Phase 3	229	229	2	20/5	24/2	52.8	PTV Dmax ≤ 150% PD	ICPRE criteria	ICPRE criteria
Pielkenrood et al. [18]	2021	Phase 2	110	55	4	8/1, 20/5, 30/10	18/1, 30/3, 35/5	50.4, 60, 59.5	PD to the visible metastasis and ≤ 50% PD to the bony compartment	ICPRE criteria	ICPRE criteria
Sprave et al. [19]	2018	Phase 2	60	60	No rules	30/10	24/1	81.6	PD at the 80% isodose	ICPRE criteria	ICPRE criteria

*BED*_10_ biological equivalent dose with α/β = 10; *cEBRT* conventional external body radiotherapy; Dmax, maximum dose; *ICPRE* international consensus pain response endpoints; *NRS* numerical rating scale; *PD* prescribed dose; *PTV* planning target volume; *SBRT* stereotactic body radiotherapy

Among the seven trials used for analysis in this study, four were assessed to have a low risk of bias, and one was assessed to have some concerns of bias (Additional file 2). Two trials were determined to have a high risk of bias owing to an undescribed randomisation procedure [17] and deviations from intended interventions [18].

### Pain response

**Fig. 2:**
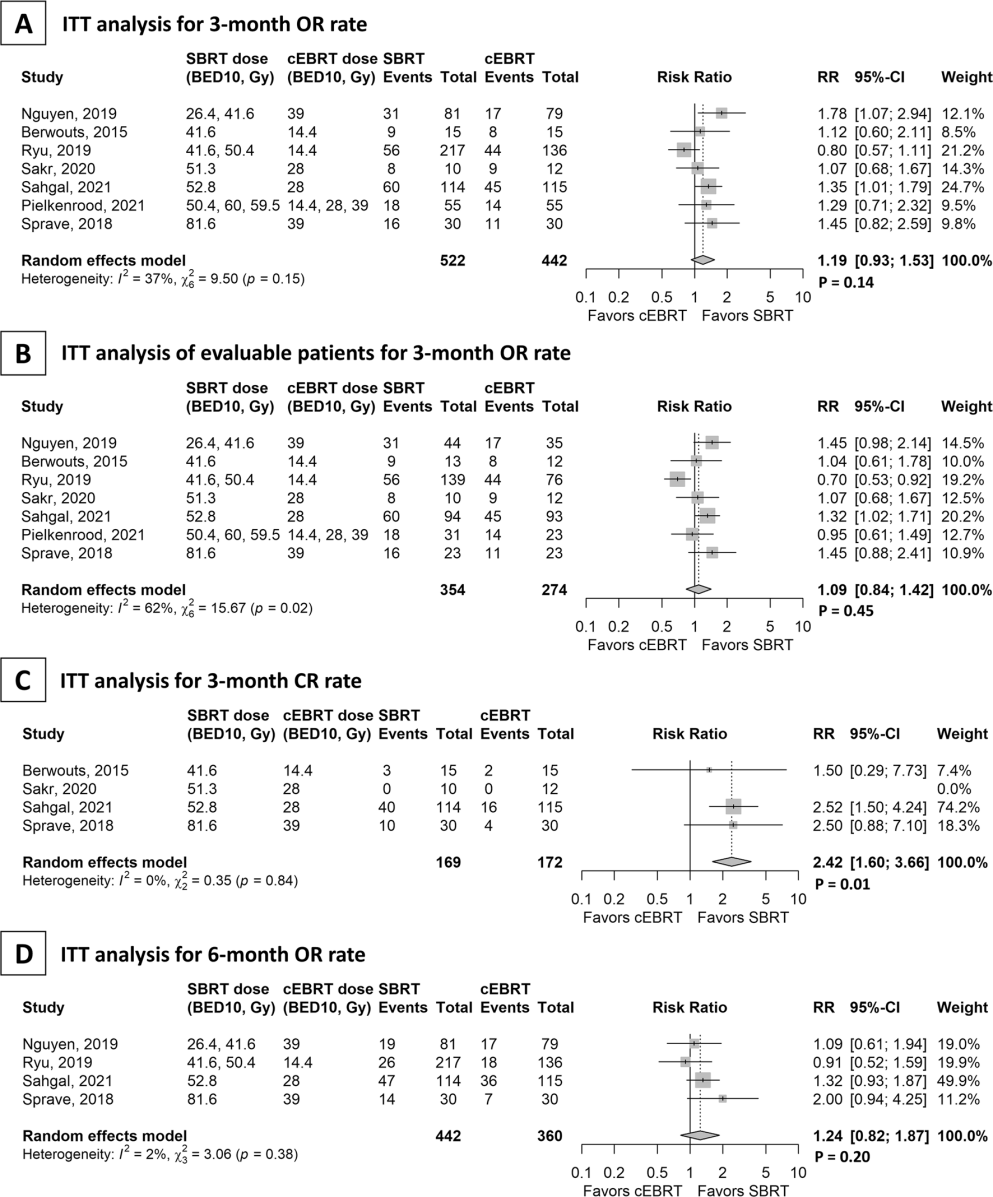
Forest plot for bone metastases. Studies are presented in ascending order of the biological equivalent dose of stereotactic body radiotherapy. **a** Overall pain response (OR) rates at 3 months in the intention-to-treat (ITT) analysis (primary outcome). **b** OR rate at 3 months in the ITT analysis of evaluable patients. **c** Complete pain response rate at 3 months in the ITT analysis. **d** OR rate at 6 months in the ITT analysis

In the seven studies included for the calculation of OR rates of bone pain at 3 months in the ITT analysis, the SBRT and cEBRT arms included 522 and 442 patients, respectively. The OR rates estimated using random-effects models were 45% (95% CI 31–60%) and 36% (95% CI 25–49%) in the SBRT and cEBRT arms, respectively. These studies showed no significant difference between the two groups (RR = 1.19; 95% CI 0.93–1.53; p = 0.14, Fig. 2A). Although Cochran’s Q test was not significant (p = 0.15), the *I*^*2*^ value (37%) indicated the possibility of moderate heterogeneity between the studies [14]. Inspection of a funnel plot found no substantial evidence of publication bias (see Additional file 3). In all seven studies, the ITT analysis of evaluable patients, including 354 and 274 patients in the SBRT and cEBRT arms, respectively, showed no significant difference in the OR rates (62% vs. 54%; RR = 1.09; 95% CI 0.84–1.42; p = 0.45; Fig. 2b). Regarding the CR rate, four studies with 169 and 172 patients in the SBRT and cEBRT arms, respectively, showed a significant difference favouring SBRT (31% vs. 13%; RR = 2.42; 95% CI 1.60–3.66; p = 0.01; Fig. 2c). In the evaluation at 6 months, four studies with 442 and 360 patients in the SBRT and cEBRT arms, respectively, showed no significant difference in the OR rates (28% vs. 21%; RR = 1.24; 95% CI 0.82–1.87; p = 0.20; Fig. 2d). ×

**Fig. 3:**
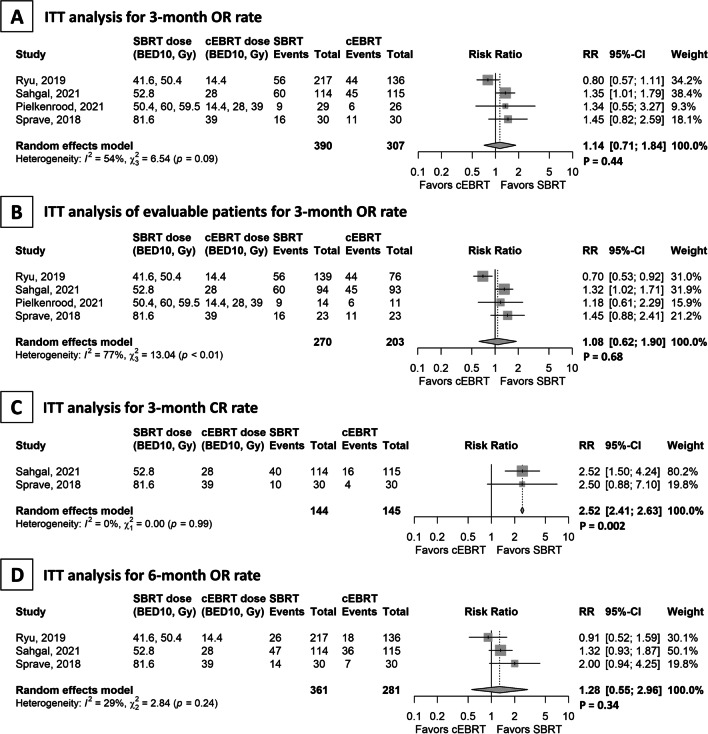
Forest plot for spinal metastases. Studies are presented in ascending order of the biological equivalent dose of stereotactic body radiotherapy. **a** Overall pain response (OR) rates at 3 months in the intention-to-treat (ITT) analysis. **b** OR rate at 3 months in the ITT analysis of evaluable patients. **c** Complete pain response rate at 3 months in the ITT analysis. **d** OR rate at 6 months in the ITT analysis

In the four studies included for the calculation of OR rates of spinal metastases at 3 months in the ITT analysis, the SBRT and cEBRT arms included 390 and 307 patients, respectively. The OR rates estimated using random-effects models were 40% (95% CI 21–62%) and 35% (95% CI 26–44%) in the SBRT and cEBRT arms, respectively. These studies showed no significant difference between the two groups (RR = 1.14; 95% CI 0.71–1.84; p = 0.44; Fig. 3A). Cochran’s Q test (p = 0.09) and the *I*^*2*^ value (54%) indicated a possibility of strong heterogeneity between the studies [14]. In the ITT analysis of evaluable patients, four studies with 270 and 203 patients in the SBRT and cEBRT arms, respectively, showed no significant difference in the OR rates (57% vs. 52%; RR = 1.08; 95% CI 0.62–1.90; p = 0.68; Fig. 3b). Regarding the CR rate for spinal metastases, two studies with 144 and 145 patients in the SBRT and cEBRT arms, respectively, showed a significant difference favouring SBRT (35% vs. 14%; RR = 2.52; 95% CI 2.41–2.63; p = 0.002; Fig. 3c). In the evaluation at 6 months, three studies with 361 and 281 patients in the SBRT and cEBRT arms, respectively, showed no significant difference in the OR rates (30% vs. 21%; RR = 1.28; 95% CI 0.55–2.96; p = 0.46; Fig. 3d).

### Adverse events

Six studies provided information on AEs post radiotherapy (Table 2) [10, 11, 15, 16, 18, 19]. Radiation myelopathy was not confirmed in any trial. Pathological fractures were encountered in 5.6% (24/427) of patients in the SBRT arm and 7.5% (26/345) of patients in the cEBRT arm. Vertebral compression fractures, which are characteristic AEs of spine SBRT, were confirmed in 6.6% (22/331) of patients in the SBRT arm and 10.0% (25/251) of patients in the cEBRT arm. The incidence of other severe AEs (≥ grade 3) was 5.8% (29/497) and 5.1% (21/415) in the SBRT and cEBRT arms, respectively.

**Table 2:** Adverse events and survival

Author	No. of patients (SBRT/cEBRT)	Pain flares	Pathological fractures	Neurological injuries	Others (≥ G3)	Median follow-up (months)	Overall survival (months)
Nguyen et al. [15]	81/79	Not available	SBRT: 1 cEBRT: 0	Not available	SBRT: G3 nausea, 1; G3 vomiting, 0; G3 fatigue, 8 cEBRT: G3 nausea, 4; G3 vomiting, 2; G3 fatigue, 4	Not available	SBRT: MST 6.7 cEBRT: MST 6.7
Berwouts et al. [16]	15/15	SBRT: 3 cEBRT: 1	SBRT: 1 cEBRT: 1	Not available	Not available	6	Whole: MST 8 (no difference between arms)
Ryu et al. [10]	217/136	Not available	SBRT: G1-2, 10; ≥ G3, 0 cEBRT: G1-2, 4; ≥ G3, 1	SBRT: G1-2 peripheral neuropathy, 21 cEBRT: G1-2 peripheral neuropathy, 11	SBRT: 19 cEBRT: 9	Not available	Not available
Sakr et al. [17]	10/12	Not available	Not available	Not available	Not available	Not available	Not available
Sahgal et al. [11]	114/115	SBRT: G3, 5 cEBRT: G3, 5	SBRT: G1, 11; G3, 1 cEBRT: G1, 19; G4, 1	None	SBRT: G3 dysphagia, 1 cEBRT: G3 nausea, 1; G3 fatigue, 1	6.7	SBRT: 3-m 93%, 6-m 77% cEBRT: 3-m 89%, 6-m 73%
Pielkenrood et al. [18]	55/55	Not available	Not available	Not available	None	AEs were confirmed within 3 months	SBRT: 3-m 84% cEBRT: 3-m 84%
Sprave et al. [19]	30/30	SBRT: 2 cEBRT: 0	Not available	None	None	Mean: 8.1	SBRT: MST 7.9 cEBRT: MST 7.9

*cEBRT* conventional external body radiotherapy; *G* grade; *MST* median survival time; *SBRT* stereotactic body radiotherapy; *AE* adverse event

### Health-related QOL

Six trials used a total of 12 QOL instruments to compare the two treatment groups (Table 3) [10, 11, 15, 16, 22, 23]. Seven QOL instruments showed no significant differences between the arms in all domains at all recorded time points [10, 11, 16, 23]. Two QOL instruments [11, 15] favoured SBRT and three [10, 22] favoured cEBRT. Reproducible results to confirm the superiority of either SBRT or cEBRT could not be produced.

**Table 3:** Outcomes of health-related quality of life

Author	EORTC QLQ-BM22	EORTC QLQ-C15-PAL	Others
Nguyen et al. [15]	NA	NA	A quality-life-adjusted survival (using the Q-TWiST method): significantly higher in SBRT
Berwouts et al. [16]	Painful sites: non-significantly better in SBRT (p = 0.07)	No significant differences in any domain	NA
Ryu et al. [10]	NA	NA	EQ-5D: significantly better in cEBRT (p = 0.01) FACT-G: no significant difference (p = 0.57)
Sakr et al. [17]	NA	NA	NA
Sahgal et al. [11]	No significant differences in any domain	NA	QLQ-C30: SBRT improved the financial and physical burden compared to cEBRT (p = 0.03 and p = 0.04, respectively)
Pielkenrood et al. [22]	Functional interference: significantly better at 12 weeks in cEBRT (p = 0.04)	Emotional functioning: significantly better at 8 weeks in cEBRT	NA
Sprave et al. [23]	No significant differences in any domain at 3 and 6 months	NA	EORTC QLQ FA13: no significant differences at 3 and 6 months QSC-R10: no significant differences at 3 (p = 0.25) and 6 months (p = 0.60)

*cEBRT* conventional external body radiotherapy; *EORTC QLQ*, European Organization for Research and Treatment of Cancer Quality of Life Questionnaire; *MDASI* MD Anderson Symptom Inventory; *NA* not applicable; *SBRT* stereotactic body radiotherapy; *Q-TWiST* quality-adjusted time without symptoms of disease and toxicity; *FACT-G* functional assessment of cancer therapy–general; *QSC-R10* questionnaire on stress in cancer patients

### Survival

No significant survival differences were found between the SBRT and cEBRT arms in any of the studies (Table 2).

## Discussion

Bone SBRT aims to completely control oligometastasis, relieve painful lesions, and improve neurological function in patients with epidural spinal cord compression [24]. To verify the superiority of bone SBRT over cEBRT in terms of pain palliation, we conducted a systematic review and meta-analysis of RCTs. In the primary outcome of the 3-month OR rate in ITT analysis, the OR rate of SBRT was not significantly higher than that of cEBRT. Subgroup analyses that focused on spinal metastases also did not show a significant difference.

Although the SC.24 trial showed that the pain-relieving effects of SBRT were superior to those of cEBRT [11], the present meta-analysis did not confirm this superiority. Therefore, the superior effectiveness of SBRT is uncertain, although its safety was comparable to that of cEBRT. Furthermore, SBRT is inconvenient compared to cEBRT as it requires extensive immobilisation devices, magnetic resonance imaging for delineation of the cord, more planning time, longer treatment time per fraction, additional personnel involvement, and considerable technical investment [25–27]. Additionally, the medical expenses are high. Although SBRT for painful bone metastases is considered one of the standard treatment options, we recommend that clinicians select the optimal irradiation method for each patient.

The reasons for the significant discrepancy in results between two phase III trials [10, 11] are unclear. However, the prescribed SBRT dose may have contributed to the difference (16 Gy or 18 Gy in a single fraction [BED_10_: 41.6 Gy or 50.4 Gy, respectively] [10] vs. 24 Gy in two fractions [BED_10_: 52.8 Gy] [11]). To determine the effect of SBRT dose on pain relief, we described the forest plots in ascending order of BED_10_ of SBRT dose (Fig. 2), and no clear trend was observed. Other concerns about the RTOG0631 trial include the following: (i) significant difference in performance status between groups (p = 0.02), (ii) unknown intergroup differences in tumour characteristics (bulky metastases, extent of epidural disease, and spinal instability), (iii) unknown dosimetric data of SBRT (coverage of the target and central target dose), and (iv) unknown details of AEs, including pain flares and vertebral compression fractures. Publication of the RTOG0631 trial is awaited.

The present study included trials of spinal and non-spine bone metastases (three trials involving the spine, one involving non-spine bone, and three involving both). The difference between spine and non-spine bone metastases may have induced different OR rates due to differences in dose coverage and occurrence rate of painful fractures. Spine SBRT often cannot cover the planning target volume with the prescribed dose because of overlap with the spinal cord. However, since the prescribed doses vary for each trial, the impact of dose coverage on the pain response is considered minor. Regarding painful fractures, spine SBRT is known to cause fractures at approximately 15% [28], most of which are painless [11]. Although few reports of SBRT-induced non-spine bone fractures are published, two large-scale retrospective studies suggested a low occurrence rate at 7.0–8.5% [29, 30]. Therefore, we determined that it was valid to synthesize the trials of spinal and non-spine bone metastases.

Ablative irradiation with SBRT has strong antitumor effects, resulting in a high tumour control rate [7, 9]. If this assumption holds, SBRT is expected to improve the complete pain response rate or long-term pain control. The present meta-analysis evaluated CR rate as a secondary outcome and showed a significantly high response rate to SBRT. However, interpretation of the positive results requires attention because RTOG0631, a key trial, was not included. To clarify long-term pain control, the OR rate at 6 months was calculated including the data from the RTOG0631 trial, but this analysis showed no significant difference. One of the most important issues in bone SBRT for pain palliation is the correct selection of patients. SBRT may be suitable for patients in whom cEBRT is unlikely to provide pain relief (patients with radioresistant tumours [31], previously irradiated lesions [32], and those requiring long-term pain control [5]). Verification trials focusing on these patient cohorts would be valuable.

This study has some limitations. First, there was no access to the individual patient data. Therefore, the heterogeneous population was analysed as a single population. If the population is analysed separately according to performance status, relative radiosensitivity, and severe or mild pain at baseline, patients who will benefit from SBRT may be identified. Second, moderate to strong heterogeneity was observed in the main analyses. This heterogeneity might be due to intertrial differences in the patient cohort, SBRT dose, or SBRT planning. Third, only two phase III trials were included in this study. Since the results of the two trials were definitely conflicting, the present synthetic analysis could not find a significant difference. Hence, further studies on phase III trials are required.

## Conclusions

To the best of our knowledge, this study is the first meta-analysis of RCTs comparing SBRT and cEBRT for the treatment of bone metastases. The study findings show no significant differences between SBRT and cEBRT in terms of OR, AEs, QOL, and overall survival. Subgroup analyses of spinal metastases also showed no difference in OR. Although many retrospective studies and single-arm prospective trials have reported excellent palliative effects of bone SBRT, they may have included strong selection bias because SBRT is administered to patients with better general conditions than those who receive cEBRT. Since there are currently only two results from phase III trials, further high-quality prospective trials are required to conclude whether SBRT is a better clinical choice than cEBRT.

## Supplementary Information

**Additional file 1**: PubMed search strategy. The PubMed database was searched for relevant publications on January 22, 2022. Seven studies that met all the inclusion criteria were included in this study.

**Additional file 2**: Risk of bias assessment using the Cochrane risk of bias tool. Four, one, and two trials were assessed to have a low risk, some concerns, and a high risk of bias, respectively.

**Additional file 3**: Funnel plot of studies that reported overall pain response rates. Inspection of a funnel plot found no substantial evidence of publication bias.

## Data Availability

The datasets used and/or analysed during this study are available from the corresponding author on reasonable request.
