# Supplementary material for: Stereotactic body radiotherapy versus conventional radiotherapy for painful bone metastases: a systematic review and meta-analysis of randomised controlled trials
Source: Radiat Oncol. 2022 Sep 13;17:156. doi: 10.1186/s13014-022-02128-w (PMC9472415; doi:10.1186/s13014-022-02128-w)
Supplement: Supplementary file 1 — Additional file 1: PubMed search strategy. The PubMed database was searched for relevant publications on January 22, 2022. Seven studies that met all the inclusion criteria were included in this study. [file 13014_2022_2128_MOESM1_ESM.docx]

**Additional File 1.** PubMed search strategy

| Search No. | Search strategy | Results |
| --- | --- | --- |
| 1. | bone OR bony OR bones OR skeletal OR skeleton OR osseous OR spine OR spinal OR non-spine OR non-spinal OR nonspine OR nonspinal | 2,192,086 |
| 2. | metastasis OR metastases OR metastatic OR metastatically | 1,485,342 |
| 3. | 1. AND 2. | 162,823 |
| 4. | radiation therapy OR radiation OR radiotherapy OR irradiation OR radiotherapeutic OR RT | 1,477,308 |
| 5. | stereotactic | 30,934 |
| 6. | 4. AND 5. | 19,609 |
| 7. | 3. AND 6. | 1,245 |
| 8. | 7. AND Filters: Randomized Controlled Trial | 23 |
