# Supplementary material for: Stereotactic body radiotherapy versus conventional radiotherapy for painful bone metastases: a systematic review and meta-analysis of randomised controlled trials
Source: Radiat Oncol. 2022 Sep 13;17:156. doi: 10.1186/s13014-022-02128-w (PMC9472415; doi:10.1186/s13014-022-02128-w)
Supplement: Supplementary file 2 — Additional file 2: Risk of bias assessment using the Cochrane risk of bias tool. Four, one, and two trials were assessed to have a low risk, some concerns, and a high risk of bias, respectively. [file 13014_2022_2128_MOESM2_ESM.docx]

**Additional File 2.** Risk of bias assessment using the Cochrane risk of bias tool

| Publication | Randomisation process | Deviations from intended interventions | Missing outcome data | Measurement of the outcome | Selection of the reported result | Overall bias |
| --- | --- | --- | --- | --- | --- | --- |
| Nguyen et al. [15] | Low | Low | Low | Low | Some concerns | Some concerns |
| Berwouts et al. [16] | Low | Low | Low | Low | Low | Low |
| Ryu et al. [10] | Low | Low | Low | Low | Low | Low |
| Sakr et al. [17] | Some concerns | Some concerns | Some concerns | Low | Low | High |
| Sahgal et al. [11] | Low | Low | Low | Low | Low | Low |
| Pielkenrood et al. [18] | Low | High | Low | Low | Low | High |
| Sprave et al. [19] | Low | Low | Low | Low | Low | Low |
