# Supplementary figures and images for: Stereotactic body radiotherapy versus conventional radiotherapy for painful bone metastases: a systematic review and meta-analysis of randomised controlled trials
Source: Radiat Oncol. 2022 Sep 13;17:156. doi: 10.1186/s13014-022-02128-w (PMC9472415; doi:10.1186/s13014-022-02128-w)

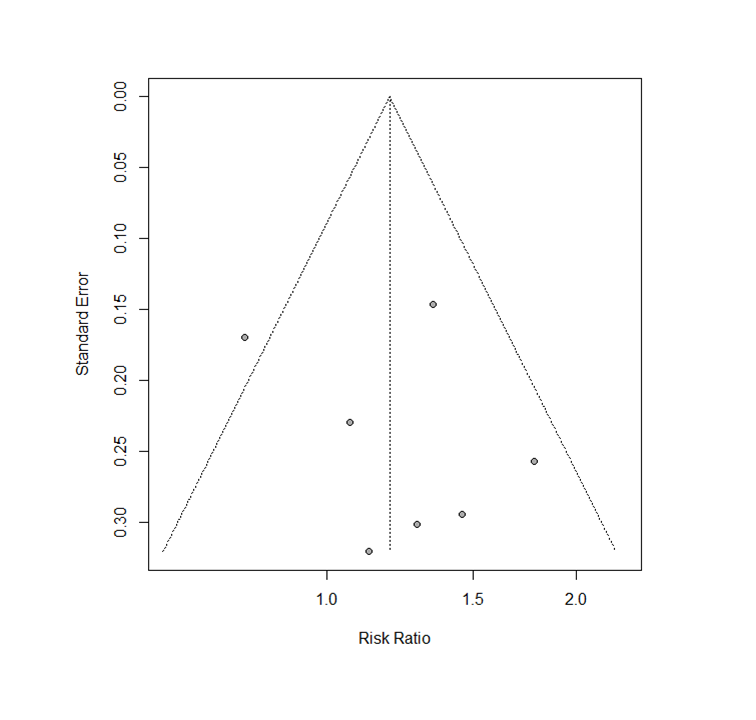

Supplement: Supplementary file 3 — Additional file 3: Funnel plot of studies that reported overall pain response rates. Inspection of a funnel plot found no substantial evidence of publication bias. [file 13014_2022_2128_MOESM3_ESM.tif]
